# Emergency healthcare delivery: determinants and systemic barriers at Wassa Amenfi East District Hospital, Ghana

**DOI:** 10.1186/s12245-026-01246-6

**Published:** 2026-05-12

**Authors:** Tonnies Abeku Buckman, Joseph Teye Nuertey, Esther Acquah, Samuel Asamoah Sakyi, Lawrence Owusu Brenya, Akwasi Minnah Addei, Andy Opoku Boateng, Maxwell Hurbert Antwi, Frank Enock Gyamfi, Michael Ofoe Adinku

**Affiliations:** 1 Department of Medical Laboratory Science, KAAF University, Fetteh-Kakraba, Gomoa -East, Ghana; 2 Department of Physician Assistant, KAAF University, Fetteh-Kakraba, Gomoa - East, Ghana; 3 Darmang CHIPS, Wassa Amenfi East District, Wassa Amenfi East, Ghana; 4 Department of Molecular Medicine, School of Medicine and Dentistry, Kwame Nkrumah University of Science and Technology, Kumasi, Ghana; 5 Department of Biochemistry and Molecular Biology, Louisiana State University, Shreveport, USA; 6 Department of Theoretical and Applied Biology, Kwame Nkrumah University of Science and Technology, Kumasi, Ghana; 7 Garden City University, Kumasi, Ghana; 8 Department of Medical Laboratory Science, Koforidua Technical University, Koforidua, Ghana; 9 Holy Family Hospital, Berekum, Ghana; 10 Department of Surgery, School of Medicine and Dentistry, Kwame Nkrumah University of Science and Technology, Kumasi, Ghana

**Keywords:** Service delivery challenges, Emergency healthcare, Healthcare infrastructure, Rural health systems

## Abstract

**Background:**

Despite the critical role emergency healthcare systems play in safeguarding lives, there remains a significant disconnect between service availability and service effectiveness in many rural settings. This study investigates systemic inefficiencies in emergency healthcare delivery at Wasa Amenfi East District Hospital, Ghana, highlighting the disconnect between policy frameworks and operational realities in a rural setting.

**Method:**

A descriptive survey design was employed to assess the effectiveness and accessibility of emergency healthcare services at Wasa Amenfi East District Hospital. Data were collected from a purposive sample of 200 participants, including healthcare professionals and patients, using structured questionnaires. The instrument captured quantitative insights on service utilization, policy awareness, infrastructure, and perceived quality of care. Descriptive statistics and chi-square tests were used to analyze relationships between demographic variables and policy awareness, with significance set at *p* < 0.05.

**Results:**

Findings reveal high utilization of emergency services (86.5%) but widespread dissatisfaction with service quality, responsiveness, staffing, and equipment availability. Key challenges include inadequate staffing (69.5%), drug shortages (71.5%), poor road infrastructure (76%), and bureaucratic delays (74%). Although 71% of respondents were aware of emergency healthcare policies, only 29.6% perceived their full implementation, and just 42.5% believed these policies improved access to healthcare. Education level was significantly associated with policy awareness (*p* = 0.049).

**Conclusion:**

The study underscores the need for strategic reforms in workforce capacity, infrastructure, and policy enforcement, advocating for community-inclusive approaches to enhance emergency care responsiveness and equity.

**Supplementary Information:**

The online version contains supplementary material available at 10.1186/s12245-026-01246-6.

## Introduction

Emergency healthcare systems are the pulse points of any medical infrastructure, designed to respond with precision and urgency when lives hang in the balance [1–5]. They represent the convergence of clinical expertise, logistical coordination, and infrastructural readiness, where every second can determine survival. As Sagan and Richardson [4] observe, emergency medical services are not only the first point of contact in life-threatening situations but also serve as a sentinel for broader health system vulnerabilities [4]. The World Health Organisation reinforces this view, describing emergency care as “an integrated platform for delivering accessible, quality, and time-sensitive health care services for acute illness and injury.” [5].

Yet in many developing regions, this vital platform is compromised by systemic inefficiencies. Infrastructural deficiencies, bureaucratic delays, and inconsistent policy implementation undermine the effectiveness of emergency care [3, 5, 6]. In their assessment of Sub-Saharan Africa, Renee and colleagues found that no surveyed hospital met the minimum WHO standards for emergency infrastructure [3]. The challenges intensify in rural settings, where socioeconomic disparities, limited public awareness, and logistical constraints converge to form a fragile and often overwhelmed framework of care [3, 5, 6]. In Ghana, Asiedu-Berkoe et al., [2] identified poor coordination, inadequate infrastructure, and weak policy execution as persistent barriers to emergency preparedness [2]. Similarly, Asamoah et al., [1] documented systemic delays caused by poor road networks, insufficient ambulance services, and bureaucratic hospital procedures, arguing that these health system failures are major contributors to late interventions and adverse outcomes [1].

Despite the critical role emergency healthcare systems play in safeguarding lives, there remains a significant disconnect between service availability and service effectiveness in many rural settings. This disconnect is particularly pronounced in districts such as Wassa Amenfi East in the Western North Region of Ghana [1, 2].

Wassa Amenfi East District Hospital is the principal government health facility serving the Wassa Amenfi East District. Located in a predominantly rural setting, the hospital provides primary and secondary level healthcare, including emergency services, to a largely agrarian population with limited access to higher-tier referral facilities. The rural character of the district is significant: road networks are poorly developed, ambulance coverage is limited, and socioeconomic constraints reduce health-seeking behaviour, all of which compound the challenges of delivering timely emergency care [1, 3].

Ghana’s National Health Policy MOH [7], sets out explicit commitments to ensuring accessible, quality emergency healthcare across all levels of the health system, including district hospitals. These commitments encompass 24/7 emergency service availability, defined staffing norms, standardised triage and referral protocols, and regular equipment maintenance. However, Ministry of Health acknowledges that policy implementation is often partial, and that community awareness and operational capacity vary considerably across districts, particularly in rural and peri-urban settings MOH [7]. At Wassa Amenfi East District Hospital, preliminary data from routine hospital records and informal engagement with frontline staff suggest that, while emergency services are structurally present and the aforementioned policy frameworks nominally apply, their implementation is inconsistent and their impact on care delivery remains limited [1, 2]. Observed challenges include inadequate staffing levels, intermittent drug and equipment availability, and administrative delays in patient processing. These preliminary findings, which this study empirically examined, are consistent with documented patterns at comparable district-level facilities across Ghana [2]. This gap between infrastructural intent and experiential reality, where policy awareness does not translate into operational efficiency and where 24/7 service availability is undermined by staffing shortages, equipment deficits, and administrative delays, warrants systematic investigation. Existing literature has extensively documented national-level challenges in emergency healthcare across sub-Saharan Africa [1–3, 8, 9], yet few studies offer a granular, district-level analysis that incorporates the perspectives of both patients and providers. Such a district-level focus is important because aggregate national data can obscure specific resource, infrastructure, and governance deficits that characterise individual facilities. Equally, capturing perspectives from both healthcare providers and patients is important: providers reveal the operational constraints and systemic barriers shaping care delivery, while patients illuminate whether services are accessible, responsive, and of adequate quality in practice. Together, these perspectives enable a more complete diagnosis of system failures and a more targeted set of reform recommendations [5, 10]. This study addresses that gap by empirically examining sociodemographic factors, service utilisation trends, stakeholder perceptions, and policy awareness within the Wassa Amenfi East context. The specific objectives were to: (i) assess the current state of emergency healthcare service delivery at Wassa Amenfi East District Hospital; (ii) identify the key systemic and logistical challenges affecting timely emergency care; (iii) examine the level of awareness and perceived implementation of emergency healthcare policies among staff and patients; and (iv) determine the association between sociodemographic characteristics and policy awareness. By addressing these objectives, the study provides a crucial understanding of the systemic barriers to timely, equitable, and quality emergency care, and its findings may inform a reform strategy that prioritises workforce readiness, technological equipping, effective communication, and participatory governance.

## Methods

### Study design

This study utilized a descriptive survey design to systematically assess the state of emergency healthcare delivery at Wassa Amenfi East District Hospital. This approach allowed for the collection of quantitative data, providing a broad overview of the state of emergency services, key challenges, and policy impacts at the study site [11, 12]. 

### Population of the study

The target population for this study comprised all healthcare workers (including doctors, nurses, and emergency staff) and patients aged 18 years and above at Wassa Amenfi East District Hospital, as they were directly involved in or affected by emergency healthcare services. Patients below 18 years of age were excluded. Given that the data collection instrument was a self-administered written questionnaire, literacy in English or the local language (with researcher assistance for translation) was a requirement for patient participation, as described further in Section “Data collection instruments”.

### Sample and sampling procedure

The sample for this study was selected using a simple random sampling technique, following the approach outlined by Starnes, Yates, and Moore [13]. This method ensured that every healthcare professional and patient within the hospital’s emergency unit had an equal chance of being selected, minimizing selection bias. The target population comprised healthcare workers and patients utilizing emergency services. The sample size was calculated using Taro Yamane’s (1967) formula: n = N / (1 + Ne²), where N is the total population and e is the margin of error (set at 5%). Based on hospital records at the time of the study, the total population comprised approximately 150 healthcare workers and 250 patients who had utilized emergency services in the preceding six months, giving a combined population (N) of 400. Applying the formula yielded a minimum sample size of approximately 200 participants. Proportional allocation was used to determine the number drawn from each subgroup: 75 healthcare workers and 125 patients.

### Data collection instruments

The primary tool for data collection in this study was a structured questionnaire, chosen for its suitability for collecting standardised data from a large number of participants. As noted in Section “Population of the study”, participation was limited to individuals who could read and understand the questionnaire independently or with minimal assistance, consistent with the eligibility criterion of functional literacy. The instrument was cost-effective, easy to administer, and ensured consistency in the questions asked, facilitating uniform data collection. The structured questionnaire also guaranteed respondent anonymity, which encouraged honest and open responses without fear of judgment or stigmatization. Responses were captured using a five-point Likert-type agreement scale (ranging from “Strongly Agree” to “Strongly Disagree”) for attitudinal and perception items, and categorical or frequency-based response options for factual and demographic items. The questionnaire consisted of five sections: Section A gathered demographic data such as age, sex, and educational background. Section B assessed the state of emergency healthcare services at the Wassa Amenfi East District Hospital. Section C identified key challenges affecting the timely delivery of emergency healthcare services. Section D evaluated the impact of healthcare policies on the accessibility and quality of these services. This structured approach ensured comprehensive and accurate data collection.

#### Validity and reliability of the instruments

To ensure the accuracy and reliability of the study instruments, a pre-test study was conducted to assess validity, measuring how well an indicator captured the intended concept [12]. The findings, as highlighted by Bryman [11], were used to refine the instruments before distribution. Following Cooper and Schindler [14], pre-testing was carried out to identify and correct potential flaws in the research design. Also referred to as pilot testing, this process aimed to enhance the questionnaire’s validity and reliability. A total of ten participants were involved in the pilot study, but were excluded from the final analysis to prevent bias. To improve the response rate, participants were given self-administered hard copies, which were retrieved the next day. Reliability was assessed using Cronbach’s alpha, with a value of 0.70 or higher indicating an acceptable level of internal consistency.

### Data collection procedure

The data collection procedure for evaluating emergency healthcare at Wasa Amenfi East District Hospital involved administering a structured questionnaire (supplementary file). Ethical approval for the study was obtained from the Institutional Review Board of Komfo Anokye Teaching Hospital (Ref: KAAF/IRB/AP/8/25) prior to data collection. An introductory letter from KAAF University was also obtained to formally introduce the study and secure the cooperation of hospital management and staff. Prior to questionnaire distribution, the lead researcher personally explained the study’s objectives to participants in either English or the local language as appropriate. This was done individually, in a private setting away from clinical staff or other patients, to preserve confidentiality and avoid social desirability bias. Participation was entirely voluntary, and no inducements were offered. The questionnaire was self-administered in hard copy form; participants completed it independently and returned it directly to the researcher in a sealed envelope. Questionnaires were coded numerically, no names or personal identifiers were recorded, so that individual responses could not be traced back to participants. Completed questionnaires were stored in a locked cabinet accessible only to the research team and were not shared with hospital management or any other third party. No personal or pseudonymised information beyond what was captured on the questionnaire was collected.

### Data analysis

The data analysis for the study was conducted using IBM SPSS Statistics (version 26). Descriptive statistics, including means, frequencies, and percentages, were used to summarize demographic characteristics and general trends in emergency service delivery. Chi-square tests were used to assess associations between categorical variables, for example, between education level and policy awareness, between gender and satisfaction with emergency services, and between occupation and perceived quality of care. Binary logistic regression analysis was performed to examine the influence of predictor variables (such as staff availability, waiting time, and availability of logistics) on binary outcome variables (such as satisfaction with emergency care [satisfied vs. not satisfied] and perceived accessibility [accessible vs. not accessible]). For Likert-scale items used as predictors, responses were dichotomised (e.g., agree/strongly agree vs. neutral/disagree/strongly disagree) prior to inclusion in the regression models. Each predictor was first examined in a univariable model, and variables with a p-value of < 0.25 at univariable level were entered into the multivariable model. Odds ratios (ORs) and 95% confidence intervals (CIs) were reported. This combination of descriptive and inferential statistics provided both a comprehensive summary and deeper insight into the factors affecting emergency healthcare service delivery.

## Results

### Sociodemographic features of study participants

The study involved 200 participants with a wide age distribution, the majority (30.5%) being within the 26–35year age group, followed by 22.5% aged 36–45 years, and 19.5% aged 18–25 years. Only 8.5% were under 18, and 8% were above 55 years. Males constituted a slight majority, accounting for 55.5% of the participants, while females made up 44.5%. In terms of educational attainment, nearly half (45.5%) of the participants had tertiary education, and 26% had completed senior high school, whereas a minority had no formal education (10%) or only primary education (6.5%). Occupationally, government workers represented the largest group (38.5%), followed by farmers (16.5%), artisans (16%), students (15%), and traders (14%). These findings indicate a relatively young, educated, and professionally diverse study population, with a slight male predominance (Table 1).

**Table 1:** Table showing the sociodemographic features of study participants

Variable	Frequency (*n* = 200)	Percentage (%)
**Age**		
Under 18 years	17	8.5
18–25 years	39	19.5
26–35 years	61	30.5
36–45 years	45	22.5
46–55 years	22	11
Above 55 years	16	8
**Gender**		
Male	111	55.5
Female	89	44.5
**Education Level**		
No Formal education	20	10
Primary education	13	6.5
Junior High School	24	12
Senior High School	52	26
Tertiary education	91	45.5
**Occupation**		
Student	30	15
Farmer	33	16.5
Trader	28	14
Artisan	32	16
Government worker	77	38.5

### Current state of emergency health services

The assessment of emergency health services revealed mixed perceptions among the 200 respondents. While 55% (combining “agree” and “strongly agree”) affirmed that the emergency unit is accessible 24/7, only 34.5% believed that emergency staff respond promptly, and an even lower 29% agreed that there are enough qualified healthcare personnel. Furthermore, only 32% felt that patients are attended to without delays, while a significant 47.5% disagreed or strongly disagreed. Regarding equipment, just 27.5% believed the emergency unit is well-equipped, while 52% disagreed. Similarly, 29% affirmed the functionality of ambulance services, in contrast to 51.5% who disagreed. Although 37.5% considered the hospital environment clean and conducive, 38% disagreed. Effective communication was acknowledged by 38.5% of participants, while 39.5% disagreed. Drug and treatment delivery in emergencies was viewed positively by 31.5%, but 45% reported otherwise. Finally, only 31.5% rated the overall quality of emergency services as satisfactory, while 42.5% expressed dissatisfaction. These results indicate considerable concerns regarding the responsiveness, staffing, resources, and overall quality of emergency healthcare services (Table 2).

**Table 2:** Table displaying the current state of emergency health services

Variable	Frequency (*n* = 200)	Percentage (%)
**The emergency unit is accessible at all hours (24/7)**		
Strongly agree	31	15.5
Agree	79	39.5
Neutral	40	20
Disagree	27	13.5
Strongly disagree	23	11.5
**Emergency healthcare staff respond promptly to incoming cases.**		
Strongly agree	14	7
Agree	55	27.5
Neutral	43	21.5
Disagree	42	21
Strongly disagree	46	23
**There are enough qualified doctors and nurses in the emergency unit.**		
Strongly agree	9	4.5
Agree	49	24.5
Neutral	37	18.5
Disagree	55	27.5
Strongly disagree	50	25
**Patients are attended to without unnecessary delays.**		
Strongly agree	11	5.5
Agree	53	26.5
Neutral	41	20.5
Disagree	61	30.5
Strongly disagree	34	17
**The emergency unit is well-equipped with essential medical tools and supplies.**		
Strongly agree	12	6
Agree	43	21.5
Neutral	41	20.5
Disagree	52	26
Strongly disagree	52	26
**Ambulance services are available and functional for emergency cases.**		
Strongly agree	14	7
Agree	44	22
Neutral	39	19.5
Disagree	58	29
Strongly disagree	45	22.5
**The hospital environment is clean and conducive for emergency care.**		
Strongly agree	12	6
Agree	63	31.5
Neutral	49	24.5
Disagree	43	21.5
Strongly disagree	33	16.5
**There is effective communication between staff and patients in emergency situations.**		
Strongly agree	14	7
Agree	63	31.5
Neutral	44	22
Disagree	37	18.5
Strongly disagree	42	21
**Drugs and treatment are provided promptly in emergency cases.**		
Strongly agree	17	8.5
Agree	46	23
Neutral	47	23.5
Disagree	46	23
Strongly disagree	44	22
**The quality of emergency healthcare services at the hospital is satisfactory.**		
Strongly agree	16	8
Agree	47	23.5
Neutral	52	26
Disagree	48	24
Strongly disagree	37	18.5

### Involvement of participants in emergency health care

**Fig. 1:**
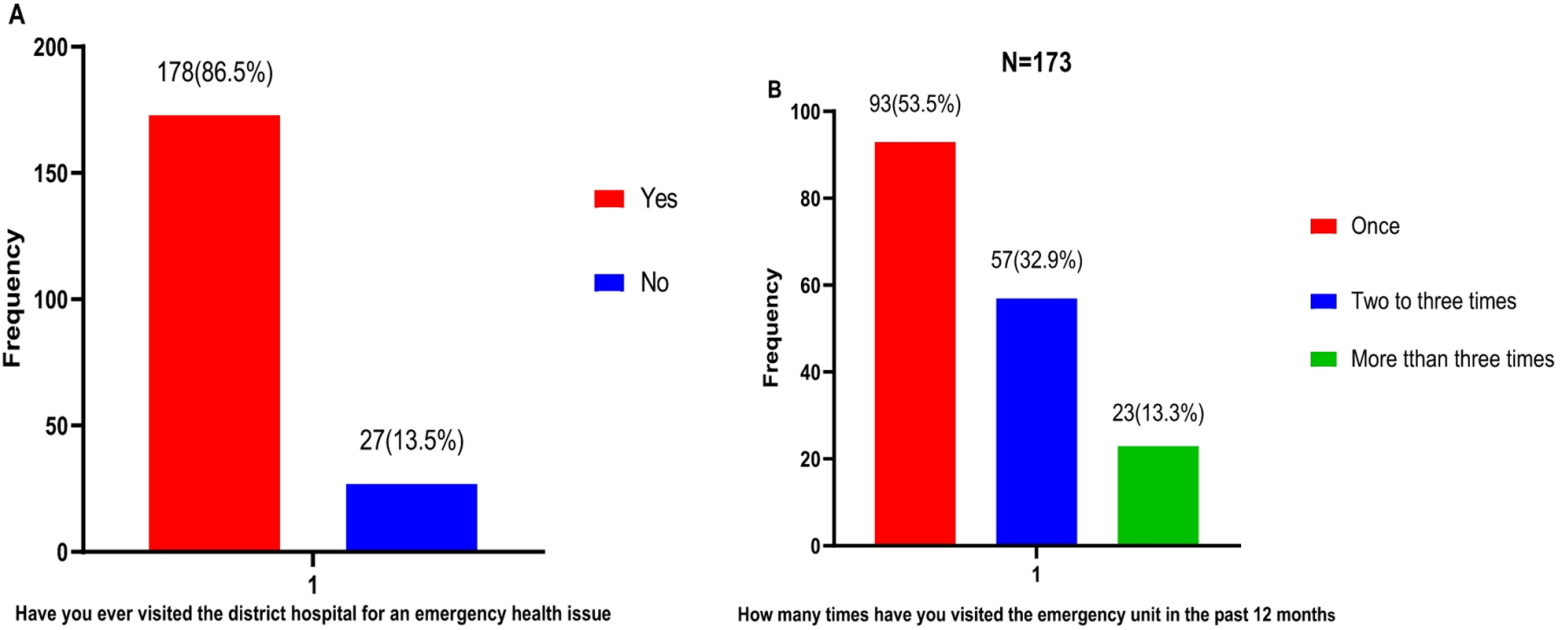
Figure showing the involvement of participants in emergency health care. **A** demonstrates the question whether one has ever visited the district hospital for emergency health issues. **B** illustrates the number of times that one has visited the emergency unit in the past 12 months

The results show that a significant majority of respondents (86.5%) reported having visited the district hospital for an emergency health issue, indicating high utilization of emergency services. Among those who had visited, more than half (53.8%) had done so only once in the past 12 months, suggesting that for many, emergency visits were infrequent or situational. However, 32.9% had visited two to three times, and 13.3% had visited three or more times, reflecting a notable proportion of individuals with repeated emergency care needs. This pattern highlights both the reliance on and the potential burden placed on emergency health services at the district hospital (Fig. 1).

### Challenges affecting the timely delivery of emergency healthcare services

The results indicate that a range of systemic and infrastructural challenges significantly affect the timely delivery of emergency healthcare services. A majority of respondents agreed or strongly agreed that inadequate staffing (69.5%), shortages of essential emergency drugs and supplies (71.5%), and lack of functioning medical equipment (71.5%) were major contributors to delays. Additionally, limited ambulance availability (72.5%) and poor road conditions (76%) were also widely recognized as critical obstacles to effective emergency referrals and patient arrival. Bureaucratic admission processes (74%), inadequate staff training (70%), power outages (71%), overcrowding in emergency units (70%), and financial constraints (72%) further compound the difficulties, reflecting a deeply strained emergency care system (Table 3).

**Table 3:** Table showing the challenges affecting the timely delivery of emergency healthcare services

Variable	Frequency (*n* = 200)	Percentage (%)
**Inadequate staffing contributes to delays in emergency response**		
Strongly agree	72	36
Agree	67	33.5
Neutral	38	19
Disagree	16	8
Strongly disagree	7	3.5
**Shortage of essential emergency drugs and supplies delays treatment.**		
Strongly agree	44	22
Agree	99	49.5
Neutral	32	16
Disagree	16	8
Strongly disagree	9	4.5
**Lack of functioning medical equipment affects timely diagnosis and treatment.**		
Strongly agree	52	26
Agree	91	45.5
Neutral	33	16.5
Disagree	16	8
Strongly disagree	8	4
**Limited availability of ambulance services affects emergency referrals.**		
Strongly agree	52	26
Agree	93	46.5
Neutral	36	18
Disagree	13	6.5
Strongly disagree	6	3
**Poor road conditions and transportation difficulties delay patients’ arrival at the hospital.**		
Strongly agree	58	29
Agree	94	47
Neutral	24	12
Disagree	20	10
Strongly disagree	4	2
**Emergency cases are sometimes delayed due to bureaucratic admission processes.**		
Strongly agree	40	20
Agree	108	54
Neutral	27	13.5
Disagree	16	8
Strongly disagree	9	4.5
**Inadequate training of healthcare workers affects efficiency in handling emergency cases.**		
Strongly agree	51	25.5
Agree	89	44.5
Neutral	29	14.5
Disagree	19	9.5
Strongly disagree	12	6
**Power outages occasionally disrupt emergency care services.**		
Strongly agree	53	26.5
Agree	89	44.5
Neutral	36	18
Disagree	17	8.5
Strongly disagree	5	2.5
**Overcrowding in the emergency unit causes delays in attending to patients.**		
Strongly agree	48	24
Agree	92	46
Neutral	29	14.5
Disagree	23	11.5
Strongly disagree	8	4
**Financial constraints limit the hospital’s capacity to provide timely emergency care.**		
Strongly agree	44	22
Agree	100	50
Neutral	29	14.5
Disagree	18	9
Strongly disagree	9	4.5

### Awareness of policies in the emergency healthcare services

The findings reveal that a majority of respondents (71%) are aware of national or hospital-level policies guiding emergency care delivery, indicating a relatively high level of policy awareness among the population. However, perceptions of policy implementation were mixed, with only 29.6% reporting full implementation, while the majority (52.8%) believed policies were only partially implemented, and 17.6% were unsure or saw no implementation at all. When asked about the impact of these policies, 42.5% felt they had improved access to emergency services, although a significant proportion (38.5%) were uncertain. Regarding quality improvement, 68.5% of respondents believed that policies had either greatly (28%) or somewhat (40.5%) improved the quality of emergency healthcare, while 31.5% were unsure or reported no improvement. These results suggest that while awareness of emergency care policies is relatively strong, their perceived implementation and impact on service access and quality remain limited and inconsistent (Table 4).

**Table 4:** Table displaying the awareness of policies in the emergency healthcare services

Variable	Frequency (*n* = 200)	Percentage (%)
**Are you aware of any national or hospital-level policies guiding emergency care delivery**		
Yes	142	71
No	58	29
**If yes**,** how well are these policies implemented**		
Fully	42	29.6
Partially	75	52.8
Not Sure	16	11.3
Not at All	9	6.3
**Have healthcare policies improved access to emergency services at this hospital**		
Yes	85	42.5
No	38	19
Not sure	77	38.5
**Have healthcare policies improved the quality of emergency services delivered**		
Greatly	56	28
Somewhat	81	40.5
Not Sure	44	22
Not at All	19	9.5

### Association between sociodemographic features of participants and awareness of policies in the emergency healthcare services

The analysis in explores the association between participants’ sociodemographic characteristics and their awareness of policies guiding emergency healthcare services. Out of 200 participants, 71% were aware of such policies. Awareness varied across age groups, with the highest awareness among those aged 26–35 years (32.4%) and 36–45 years (22.5%), while the lowest was observed in those above 55 years (6.3%) and under 18 (8.5%). However, these differences were not statistically significant (*p* = 0.1). Gender was also not significantly associated with awareness (*p* = 0.707), with 56.3% of aware participants being male and 43.7% female. Education level showed a statistically significant association with awareness (*p* = 0.049). Specifically, 50.7% of those aware had tertiary education compared to only 32.8% of the unaware group, while individuals with no formal education accounted for just 7.7% of those aware versus 15.5% among the unaware. Regarding occupation, although not statistically significant (*p* = 0.173), government workers formed the largest proportion among both aware (38.7%) and unaware (37.9%) participants, followed by artisans (19.7% aware vs. 6.9% unaware). These findings suggest that higher education levels may positively influence awareness of emergency healthcare policies, while other sociodemographic factors showed no significant associations (Table 5).

**Table 5:** Association between sociodemographic features of participants and awareness of policies in the emergency healthcare services

Variable	Awareness of National or Hospital-level policies guiding emergency care delivery		*P*-value
	Yes	No	
**Age**			0.100
Under 18 years	12(8.5%)	5(8.6%)	
18–25 years	23(16.2%)	16(27.6%)	
26–35 years	46(32.4%)	15(25.9%)	
36–45 years	32(22.5%)	13(22.4%)	
46–55 years	20(14.1%)	2(3.4%)	
Above 55 years	9(6.3%)	7(12.1%)	
**Gender**			0.707
Male	80(56.3%)	31(53.4%)	
Female	62(43.7%)	27(46.6%)	
**Education Level**			**0.049**
No Formal education	11(7.7%)	9(15.5%)	
Primary education	6(4.2%)	7(12.1%)	
Junior High School	16(11.3%)	8(13.8%)	
Senior High School	37(26.1%)	15(25.9%)	
Tertiary education	72(50.7%)	19(32.8%)	
**Occupation**			0.173
Student	20(14.1%)	10(17.2%)	
Farmer	20(14.1%)	13(22.4%)	
Trader	19(13.4%)	9(15.5%)	
Artisan	28(19.7%)	4(6.9%)	
Government worker	55(38.7%)	22(37.9%)	

## Discussion

This study aimed to explore the current state, challenges, and policy implications surrounding emergency healthcare services at Wasa Amenfi East District Hospital. The findings reveal a healthcare landscape characterised by high service utilisation, critical gaps in emergency care delivery, and varying awareness and implementation of supportive policies. Despite over half of the respondents (55%) acknowledging that the emergency unit operates 24/7, perceptions of quality and efficiency remain low. Only 31.5% of participants rated the overall emergency services as satisfactory. Particularly concerning were the low confidence levels in staff responsiveness (34.5%), adequacy of personnel (29%), and availability of equipment (27.5%). These figures suggest that, although infrastructure for continuous service delivery exists, the system’s functionality and reliability are hindered by operational inefficiencies. These findings align with the findings of some studies. These findings are consistent with evidence from comparable settings showing that patients report low satisfaction with emergency services, particularly regarding staff responsiveness, drug availability, and cleanliness, and that perceived service quality is often substantially lower than expected despite structural availability of services [2, 6, 14, 15] Jabber et al., (2024) also found that ED overcrowding, staff shortages, and workflow inefficiencies significantly reduced patient satisfaction and quality indicators [6]. Additionally, the findings reflect disparities between structural availability and service experience. The clean and conducive nature of the environment, effective communication, and drug delivery all scored below 40% in satisfaction, further revealing a service environment that lacks the consistency and readiness expected in emergency settings. According to the findings of Bobel et al. [10], structural availability alone is insufficient; patient-centred integration, communication, and staffing are critical to perceived quality [10]. This supports the idea that policy and infrastructure must align with service experience. The study highlights several systemic and logistical challenges that critically affect emergency healthcare responsiveness. Prominent among these include: Staffing shortages (69.5%), Lack of functioning equipment (71.5%), Inadequate drug supplies (71.5%), Poor Road infrastructure (76%), and Limited ambulance services (72.5%). These bottlenecks represent both in-hospital constraints and external systemic barriers that cumulatively delay diagnosis, treatment, and referrals. Importantly, bureaucratic admission processes (74%) and power outages (71%) underscore how administrative inefficiencies and infrastructural limitations obstruct even well-intentioned efforts at care delivery. The high frequency of emergency unit use, with 86.5% of respondents having visited in the past year, coupled with the challenges reported, points to a high demand for services that are under-resourced and strained. This finding supports the findings of Jabber et al., (2024), which found that emergency department overcrowding, staff shortages, and workflow inefficiencies significantly reduced patient satisfaction and quality indicators [6]. Their report again cited lack of equipment, poor communication, and administrative delays as major barriers to effective emergency care [6]. Kamba et al. [16] also reported that drug shortages, poor road infrastructure, and logistical barriers in rural emergency care [16]. Several studies also highlight how limited ambulance services, bureaucratic delays, and under-resourced facilities hinder emergency responsiveness [9, 10, 17]. This finding again aligns with the findings of Afrifa-Yamoah et al. [8], which assessed 460 healthcare facilities using a Bayesian network approach, and revealed that over 70% lacked specialized emergency personnel, 90% lacked defibrillators, and 80% had no stroke medications [8].

According to the WHO, policy effectiveness depends on clear communication, especially among populations with lower health literacy. Tailored outreach is essential to ensure equitable understanding and engagement, [5]. Although most participants (71%) reported awareness of emergency healthcare policies, their perceptions of how well these policies are implemented and effective were lukewarm. Only 29.6% believed the policies were fully operational, and just 42.5% felt they had improved access to emergency care. Nevertheless, the perceived impact of the policies on service quality appeared more positive, with 68.5% believing they had contributed beneficially to this aspect. Importantly, education level was statistically significant in determining policy awareness (*p* = 0.049), emphasising the role of educational attainment in empowering individuals to understand and engage with health systems. This agrees with the Ghana Ministry of Health’s published document that acknowledged that policy implementation is often partial, and that community awareness and education are critical to bridging the gap between policy and practice, [7]. Bobel et al. [10] also highlights that policy frameworks alone are insufficient without effective implementation and public understanding. Education and community integration are key to improving perceived service quality [10]. This suggests that policy communication and outreach may need to be tailored to reach less-educated populations more effectively.

The study’s insights underscore the urgent need for a multi-pronged approach to reform: investments in workforce capacity and equipment, streamlining of administrative processes, infrastructure development (e.g., roads, power supply), and policy implementation monitoring with community-inclusive feedback mechanisms. Notably, the finding that education level was a significant predictor of policy awareness (*p* = 0.049) has important implications for how policies are communicated. Strategies to improve emergency care outcomes should therefore include targeted health literacy programmes for less-educated populations, community engagement campaigns in local languages, and participatory governance mechanisms through which patients and community members can provide feedback on service delivery. Ultimately, for emergency care to become responsive, equitable, and reliable, both service-level improvements and strategic policy enforcement must converge. Future studies should be extended to other districts and regions to determine whether the patterns observed here are specific to Wassa Amenfi East or reflect a broader national challenge.

This study has several limitations that should be considered when interpreting its findings. First, the cross-sectional design captures a snapshot of perceptions and experiences at a single point in time; causal inferences cannot be drawn from the associations identified. Second, the study was conducted at a single facility, Wassa Amenfi East District Hospital, which limits the generalisability of findings to other district hospitals in Ghana or sub-Saharan Africa. Third, data collection relied on self-reported questionnaires, which are susceptible to social desirability bias and recall limitations, particularly for items relating to past emergency visits or policy awareness. Fourth, despite efforts to ensure functional literacy as an eligibility criterion, variations in respondents’ comprehension of certain technical items cannot be entirely excluded. Notwithstanding these limitations, this study provides a valuable empirical contribution to the sparse literature on district-level emergency care in rural Ghana.

## Conclusion

This study sheds light on the critical dynamics shaping emergency healthcare delivery at Wassa Amenfi East District Hospital. While there is evident public engagement with emergency services, the findings reveal substantial deficiencies in responsiveness, staffing, equipment, and administrative processes. Participants expressed widespread dissatisfaction with the quality and timeliness of care, citing numerous logistical and infrastructural challenges. Although awareness of emergency health policies is relatively high, their implementation and impact remain inconsistent, pointing to a gap between policy frameworks and practical realities. Importantly, the study highlights that improving emergency healthcare in the district requires more than just awareness; it demands operational commitment, systemic reform, and community-centred policy execution.

## Supplementary Information

Below is the link to the electronic supplementary material.

Supplementary Material 1

## Data Availability

The datasets during and/or analysed during the current study are available from the corresponding author upon reasonable request.
