# Supplementary material for: Emergency healthcare delivery: determinants and systemic barriers at Wassa Amenfi East District Hospital, Ghana
Source: Int J Emerg Med. 2026 May 12;19:178. doi: 10.1186/s12245-026-01246-6 (PMC13339546; doi:10.1186/s12245-026-01246-6)
Supplement: Supplementary file 1 — Supplementary Material 1 [file 12245_2026_1246_MOESM1_ESM.pdf]

**KAAF UNIVERSITY**

**FACULTY OF HEALTH AND ALLIED SCIENCES**

**DEPARTMENT OF PHYSICIAN ASSISTANT**

**Questionnaire For Participants**

The purpose of this questionnaire is to ***evaluation of emergency healthcare At District Hospital in Wasa Amenfi East***. This research is conducted in partial fulfilment of the requirement of the award of Bachelor of Degree in Physician Assistant in KAAF University. This research is conducted solely for academic purpose and respondents are therefore assured that all information provided will be treated as confidential.

**SECTION A: DEMOGRAPHIC INFORMATION**

Please tick (✔) or fill in the appropriate response. All information will be kept confidential and used strictly for research purposes.

1. Age: a. ☐ Under 18 years b. ☐ 18–25 years c. ☐ 26–35 years d. ☐ 36–45 years e. ☐ 46–55 years f. ☐ Above 55 years
2. Gender: a. ☐ Male b. ☐ Female
3. Level of Education: a. ☐ No formal education b. ☐ Primary education c. ☐ Junior High School d. ☐ Senior High School e. ☐ Tertiary education
4. Occupation: a. ☐ Student b. ☐ Farmer c. ☐ Trader d. ☐ Artisan e. ☐ Government worker f. ☐ Other (please specify): ________________________

**SECTION B: Current State of Emergency Healthcare Services**

1. Have you ever visited the district hospital for an emergency health issue?

   a. ☐ Yes b. ☐ No

2. If yes, how many times have you visited the emergency unit in the past 12 months?

   a. ☐ Once b. ☐ 2–3 times c. ☐ More than 3 times

Please indicate your level of agreement with the following statements using the scale below:

Use the following scale to rate the statement**: SD -** Strongly Disagree**, D** - Disagree **N** – Neutral, **A -** Agree and **SA -** Strongly Agree.

| **Statement** | **SD** | **D** | **N** | **A** | **SA** |
|---|---|---|---|---|---|
| 1. The emergency unit is accessible at all hours (24/7). | | | | | |
| 2. Emergency healthcare staff respond promptly to incoming cases. | | | | | |
| 3. There are enough qualified doctors and nurses in the emergency unit. | | | | | |
| 4. Patients are attended to without unnecessary delays. | | | | | |
| 5. The emergency unit is well-equipped with essential medical tools and supplies. | | | | | |
| 6. Ambulance services are available and functional for emergency cases. | | | | | |
| 7. The hospital environment is clean and conducive for emergency care. | | | | | |
| 8. There is effective communication between staff and patients in emergency situations. | | | | | |
| 9. Drugs and treatment are provided promptly in emergency cases. | | | | | |
| 10. The quality of emergency healthcare services at the hospital is satisfactory. | | | | | |

Other (please specify):

**SECTION C: Challenges Affecting the Timely Delivery of Emergency Healthcare Services in the Wasa Amenfi East District Hospital**

Use the following scale to rate the statement**: SD -** Strongly Disagree**, D** - Disagree **N** – Neutral, **A -** Agree and **SA -** Strongly Agree.

| **Statement** | **SD** | **D** | **N** | **A** | **SA** |
|---|---|---|---|---|---|
| 1. Inadequate staffing contributes to delays in emergency response. | | | | | |
| 2. Shortage of essential emergency drugs and supplies delays treatment. | | | | | |
| 3. Lack of functioning medical equipment affects timely diagnosis and treatment. | | | | | |
| 4. Limited availability of ambulance services affects emergency referrals. | | | | | |
| 5. Poor road conditions and transportation difficulties delay patients' arrival at the hospital. | | | | | |
| 6. Emergency cases are sometimes delayed due to bureaucratic admission processes. | | | | | |
| 7. Inadequate training of healthcare workers affects efficiency in handling emergency cases. | | | | | |
| 8. Power outages occasionally disrupt emergency care services. | | | | | |
| 9. Overcrowding in the emergency unit causes delays in attending to patients. | | | | | |
| 10. Financial constraints limit the hospital's capacity to provide timely emergency care. | | | | | |
